# From normal population to prediabetes and diabetes: study of influencing factors and prediction models

**DOI:** 10.3389/fendo.2023.1225696

**Published:** 2023-10-26

**Authors:** Di Gong, Xiaohong Chen, Lin Yang, Yongjian Zhang, Qianqian Zhong, Jing Liu, Chen Yan, Yongjiang Cai, Weihua Yang, Jiantao Wang

**Affiliations:** ^1^ Shenzhen Eye Hospital, Jinan University, Shenzhen, Guangdong, China; ^2^ The First Affiliated Hospital of Jinan University, Jinan University, Guangzhou, Guangdong, China; ^3^ Center of Health Management, Peking University Shenzhen Hospital, Shenzhen, Guangdong, China; ^4^ Shenzhen Eye Hospital, Jinan University, Shenzhen Eye Institute, Shenzhen, Guangdong, China

**Keywords:** prediabetes, diabetes, influencing factors, prediction model, odds ratio (OR)

## Abstract

**Objective:**

The purpose of this study is to investigate the independent influencing factors of the transition from normal population to prediabetes, and from prediabetes to diabetes, and to further construct clinical prediction models to provide a basis for the prevention and management of prediabetes and diabetes.

**Materials and methods:**

The data for this study were based on clinical information of participants from the Health Management Center of Peking University Shenzhen Hospital. Participants were classified into normal group, prediabetes group, and diabetes group according to their functional status of glucose metabolism. Spearman’s correlation coefficients were calculated for the variables, and a matrix diagram was plotted. Further, univariate and multivariate logistic regression analysis were conducted to explore the independent influencing factors. The independent influencing factors were used as predictors to construct the full-variable prediction model (Full.model) and simplified prediction model (Simplified.model).

**Results:**

This study included a total of 5310 subjects and 22 variables, among which there were 1593(30%) in the normal group, 3150(59.3%) in the prediabetes group, and 567(10.7%) in the diabetes group. The results of the multivariable logistic regression analysis showed that there were significant differences in 9 variables between the normal group and the prediabetes group, including age(Age), body mass index(BMI), systolic blood pressure(SBP), urinary glucose(U.GLU), urinary protein(PRO), total protein(TP), globulin(GLB), alanine aminotransferase(ALT), and high-density lipoprotein cholesterol(HDL-C). There were significant differences in 7 variables between the prediabetes group and the diabetes group, including Age, BMI, SBP, U.GLU, PRO, triglycerides(TG), and HDL.C. The Full.model and Simplified.model constructed based on the above influencing factors had moderate discriminative power in both the training set and the test set.

**Conclusion:**

Age, BMI, SBP, U.GLU, PRO, TP, and ALT are independent risk factors, while GLB and HDL.C are independent protective factors for the development of prediabetes in the normal population. Age, BMI, SBP, U.GLU, PRO, and TG are independent risk factors, while HDL.C is an independent protective factor for the progression from prediabetes to diabetes. The Full.model and Simplified.model developed based on these influencing factors have moderate discriminative power.

## 1 Introduction

Prediabetes, also known as impaired glucose regulation (IGR), is a pathological state where blood glucose levels are higher than normal but have not yet reached the diagnostic criteria for diabetes. It includes two types: impaired fasting glucose (IFG) and impaired glucose tolerance (IGT). IFG is fasting blood glucose between the normal range and diabetes criteria (6.1–6.9 mmol/L), while IGT is elevated blood glucose during a 2-hour oral glucose tolerance test (OGTT) but not meeting diabetes criteria (7.8–11.0 mmol/L) (1). According to a global epidemiological survey, nearly 400 million adults worldwide have prediabetes, with a prevalence of approximately 6.4% for IFG, 7.5% for IGT, and 2.4% for both IFG and IGT (2). In China, a cross-sectional survey found a high prediabetes prevalence of 35.7% among adults (3), much higher than in other regions around the world (4–6). Prediabetes is a high-risk state for developing diabetes, with an annual conversion rate of 5%-10%. However, some studies have indicated that a proportion of patients can return to normal glucose metabolism (1, 7–9). Therefore, prediabetic patients should take early control measures to prevent further development towards diabetes.

Diabetes is a chronic metabolic disease characterized by persistently high blood sugar levels, leading to damage to various organs and tissues in the body. According to the standards of the World Health Organization (WTO) and the American Diabetes Association (ADA), the diagnostic criteria for diabetes are a fasting blood sugar level ≥7.0 mmol/L or a random blood sugar level ≥11.1 mmol/L, or a blood sugar level ≥11.1 mmol/L after an oral glucose tolerance test (OGTT), or an HbA1c level of ≥6.5% measured by standardized DCCT analysis (10). Over the past 30 years, the number of diabetes patients worldwide has doubled, and there is a concerning trend of its occurrence among younger individuals (11, 12). The global prevalence of diabetes was estimated to be 9.3% (463 million people) in 2019 and is projected to increase to 10.2% (578 million people) by 2030 and 10.9% (700 million people) by 2045 (2). In populous countries, the estimated prevalence of diabetes among adults is 10.9% in China (3), 12-14% in the United States (13), and approximately 7.3% in all 15 states of India (5). Diabetes has undoubtedly become a significant challenge for global public health in the 21st century, particularly in developing countries like China and India (11).

Prediabetes, as a precursor to diabetes, can cause a range of health issues, even though IFG and IGT themselves should not be considered clinical entities. Research has shown that prediabetes is significantly associated with an increased risk of obstructive sleep apnea, composite cardiovascular disease, coronary heart disease, stroke, and all-cause mortality (14, 15). Moreover, if timely and effective measures are not taken to control blood glucose levels, prediabetes may progress into type 2 diabetes (1, 9). As a chronic metabolic disease, the long-term hyperglycemic state of diabetes patients can affect the structure and function of blood vessels through multiple pathways (16). Additionally, the hyperglycemic state can induce oxidative stress, activate the inflammatory response, and affect the coagulation system, leading to a series of pathological and physiological changes that ultimately result in the occurrence of various complications (17). According to a WHO report, diabetes has become the seventh leading cause of death in humans, with cardiovascular disease being the main cause of death and morbidity in diabetes patients (12, 18). Therefore, prevention and treatment of diabetes should be highly emphasized.

Current research indicates that prediabetes and the development of diabetes may be related to many risk factors, including age, family history, race, genetic mutations, lack of physical activity, unhealthy dietary habits, obesity, hypertension, lipoprotein, high cholesterol, and hypertriglyceridemia (1, 3, 19–22). By analyzing the disease risk factors of prediabetes and diabetes, building a clinical prediction model can help identify high-risk patients, but currently, there is no widely used prediction model in clinical practice. Wu et al. found that waist circumference, family history of diabetes, HbA1c, and fasting blood glucose levels were independently associated with the risk of prediabetes. A prediabetes prediction model was constructed by incorporating these four indicators, with an Area Under the Curve (AUC) of 0.70236, indicating a moderately low level of discrimination (23).Yokota et al. conducted a retrospective longitudinal study and found that family history of diabetes, male gender, elevated systolic blood pressure, blood glucose levels, HbA1c, and alanine aminotransferase were important independent predictors for the conversion of prediabetes to diabetes. The prediction model constructed using these variables had a Receiver Operating Characteristic (ROC) Curve of 0.8037, indicating a moderate level of discrimination (24).

Diabetes has emerged as a global public health concern, with its incidence and mortality rates steadily increasing. Prediabetes serves as a warning sign for diabetes, and early detection with effective interventions can prevent its progression, thus reducing the incidence and mortality rates of diabetes. Therefore, the purpose of this study is to conduct a statistical analysis of cross-sectional data from a population undergoing medical examinations, to explore the independent influencing factors associated with the transition from normal individuals to prediabetes and from prediabetes to diabetes. Additionally, the study aims to develop a clinical prediction model for these diseases. In this study, we aim to use blood glucose and HbA1c as the diagnostic gold standards, while considering other risk indicators as predictive factors. The goal is to identify and provide early warning of individuals at risk of prediabetes and diabetes among the population undergoing health examinations. The findings of this research have the potential to offer valuable insights into the influencing factors of prediabetes and diabetes, which could be of significance in enhancing our understanding of these conditions. Clinical practitioners may find the information helpful in making more informed decisions while diagnosing and treating diabetes patients. By identifying specific risk factors, tailored interventions may be developed to improve patient outcomes and enhance their quality of life. Furthermore, this study will serve as a crucial reference for public health workers in devising effective strategies for diabetes prevention and control, empowering them to better manage and prevent the occurrence of diabetes.

## 2 Materials and methods

### 2.1 Data source and collection

The original data for this study were collected from individuals who underwent health examinations at the Health Management Center of Peking University Shenzhen Hospital between January 2020 and March 2023. All participants underwent fasting blood glucose, random blood glucose, OGTT, and HbA1c testing according to WTO standards. The diagnostic criteria for diabetes are a fasting blood sugar level ≥7.0 mmol/L or a random blood sugar level ≥11.1 mmol/L, or a blood sugar level ≥11.1 mmol/L after oral glucose tolerance test (OGTT), or an HbA1c level of ≥6.5% measured by standardized DCCT analysis. The diagnostic criteria for prediabetes are a fasting blood glucose level in the range of 6.1-6.9 mmol/L, or a blood glucose level in the range of 7.8-11.0 mmol/L after OGTT. Participants were categorized into normal, prediabetes, and diabetes groups based on their glucose metabolism status.

### 2.2 Variable selection

Relevant literature was searched using keywords such as “prediabetes” and “diabetes” on databases including PUBMED, EMBASE, and Web of Science to determine the variables to be included in the study. The variables extracted from participants were glucose metabolism status (Status), age (Age), gender (Gender), body mass index (BMI), systolic blood pressure (SBP), urinary glucose (U.GLU), urinary protein (PRO), total protein (TP), albumin (ALB), globulin (GLB), total bilirubin (T.BIL), direct bilirubin (DB), indirect bilirubin (IB), alanine aminotransferase (ALT), aspartate aminotransferase (AST), blood urea nitrogen (BUN), serum creatinine (SCr), uric acid (UA), total cholesterol (TC), triglycerides (TG), high-density lipoprotein cholesterol (HDL-C), and low-density lipoprotein cholesterol (LDL-C) (a total of 22 variables). The extracted data were then compiled and merged into a single file based on the participants’ ID numbers.

### 2.3 Variable assignment

All categorical variables including Status, Gender, U.GLU, and PRO were assigned values. The remaining continuous variables were not assigned values. **Table 1** shows the assigned values for each variable.

**Table 1:** Variable assignment explanation.

Variable name	Meaning of variables	Type of variables	Assignment description
Status	Glucose metabolic status	Categorical variable	1=Normal
			2=Prediabetes
			3=Diabetes
Age	Age	Numerical variable	
Gender	Gender	Categorical variable	0=Female
			1=Male
BMI	BMI Index	Numerical variable	
SBP	Systolic blood pressure	Numerical variable	
U.GLU	Urine glucose	Categorical variable	0=Negative
			1=Positive
PRO	Urine protein	Categorical variable	0=Negative
			1=Positive
TP	Total protein	Numerical variable	
ALB	Albumin	Numerical variable	
GLB	Globulin	Numerical variable	
T.BIL	Total bilirubin	Numerical variable	
DB	Direct bilirubin	Numerical variable	
IB	Indirect bilirubin	Numerical variable	
ALT	Glutathione aminotransferase	Numerical variable	
AST	Glutathione transaminase	Numerical variable	
BUN	Blood urea nitrogen	Numerical variable	
SCr	Blood creatinine	Numerical variable	
UA	Uric acid	Numerical variable	
TC	Total Cholesterol	Numerical variable	
TG	Triglycerides	Numerical variable	
HDL.C	High-density lipoprotein cholesterol	Numerical variable	
LDL.C	Low-density lipoprotein cholesterol	Numerical variable	

### 2.4 Data processing and statistical analysis

This study used R 4.2.3 software for data processing and statistical analysis. Differences were considered statistically significant at P<0.05. Firstly, the complete.cases() function was used to clean missing data. Then, the summary() function was used to perform descriptive statistical analysis on the variables in the dataset. The cor() function was used to calculate the Spearman correlation coefficient between variables and the matrix plot was generated using the ggplot2 library. The glm() function was used to perform univariate regression analysis on all independent variables. Variables with statistically significant differences in univariate regression analysis were included in the multivariate regression analysis to identify independent influencing factors in the development from normal to prediabetes, and from prediabetes to diabetes. The dataset was randomly divided into training and testing sets at an 8:2 ratio. The glm() function was used to build a full variable prediction model (Full.model) and a simplified prediction model (Simplified.model) using the training set. The roc() function and function() function were used to calculate the discrimination, accuracy, precision, and recall of the Full.model and Simplified.model.

The specific explanations of the R language functions used above are as follows: complete.cases(): It is a function used for data processing to check if each row in a data frame or matrix contains complete data (without missing values). summary(): It is a function used to summarize statistical data, returning descriptive statistics for each variable, such as mean, median, minimum, maximum, and quantiles. cor(): It is a function used to calculate correlation coefficients, computing the correlation between columns of a data frame or matrix. glm(): It is a function used to fit Generalized Linear Models, allowing fitting various models, such as linear regression, logistic regression, and Poisson regression. roc(): It is a function used to compute ROC curves, which are graphical methods to evaluate the performance of binary classification models. function(): It is a keyword used to create custom functions, enabling operations based on user-defined logic and returning calculated results.

## 3 Results

### 3.1 Detection rate of prediabetes, diabetes

The health examination data of the subjects were summarized and organized, and individuals with missing variables were excluded. Ultimately, 5310 participants were included in the study and divided into three groups based on their glucose metabolism status: normal group (1593 cases, 30%), prediabetes group (3150 cases, 59.3%), and diabetes group (567 cases, 10.7%).

### 3.2 Correlation analysis between variables

**Figure 1:**
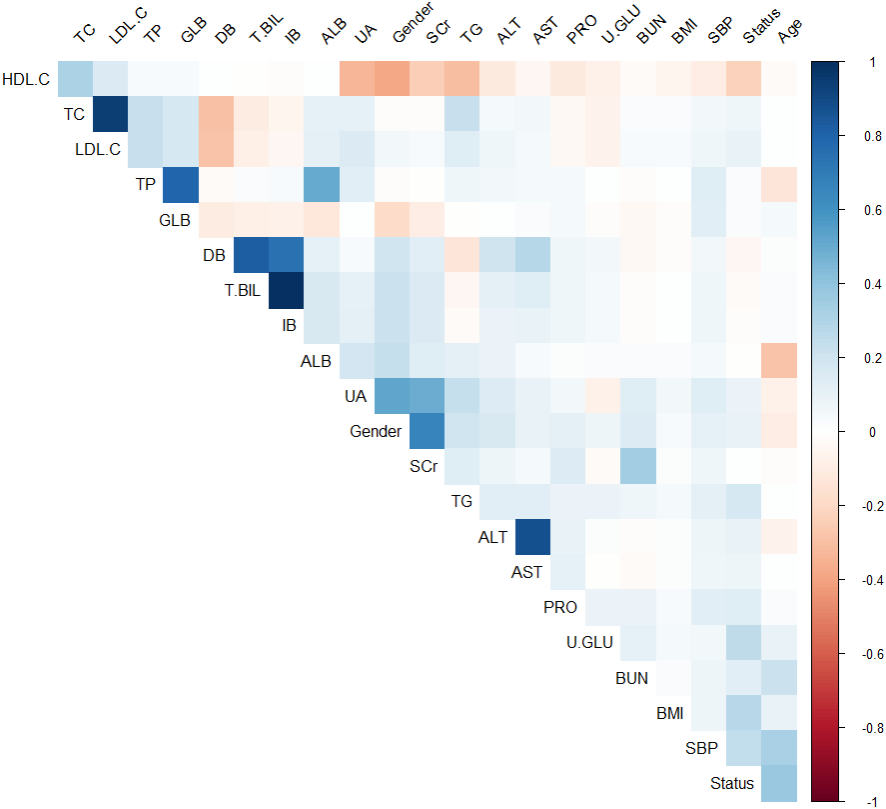
Matrix heat map based on Spearman correlation coefficients between variables (Blue represents positive correlation, while red represents negative correlation).

Calculate the Spearman correlation coefficient between each variable and use the “ggplot2” library to create a matrix heatmap. The color of each cell represents the degree of correlation between the corresponding variables. Blue represents positive correlation, while red represents negative correlation. The color depth varies according to the different correlation coefficients. The deeper the color, the stronger the correlation, while the lighter the color, the weaker the correlation. Variables with an absolute value of correlation coefficient > 0.8 are considered to have a strong correlation. The results of the correlation analysis in this study indicate that there is a strong correlation between TC and LDL.C, DB and T.BIL, IB and T.BIL, and AST and ALT, as shown in **Figure 1**.

### 3.3 Independent factors analysis of normal group and prediabetes group

Univariate regression analysis was performed on the independent variables of the normal group and prediabetes group. Variables with P < 0.05 in the Univariate regression analysis were included in the multivariate regression analysis to analyze the influencing factors of the normal population developing into prediabetes. The results of multivariate regression analysis showed that Age, BMI, SBP, U.GLU, PRO, TP, GLB, ALT, and HDL.C were the independent influencing factors for the development of prediabetes in the normal population. Among them, Age, BMI, SBP, U.GLU, PRO, TP, and ALT were independent risk factors, while GLB and HDL.C were independent protective factors, as shown in **Table 2**.

**Table 2:** Univariate and multivariate regression analysis for the normal and pre-diabetic groups.

Variable name	Univariate regression analysis			Multivariate regression analysis		
	OR	95%CI	*P*	OR	95%CI	*P*
Age	*1.09*	*1.08-1.10*	<0.001***	*1.10*	*1.09-1.11*	<0.001***
Gender(Male)	*1.40*	*1.24-1.58*	<0.001***	0.85	0.69-1.04	0.1131
BMI	*1.22*	*1.2-1.25*	<0.001***	*1.22*	*1.19-1.25*	<0.001***
SBP	*1.03*	*1.03-1.04*	<0.001***	*1.01*	*1.00-1.01*	<0.001***
U.GLU(Positive)	*6.59*	*3.05-14.24*	<0.001***	*4.23*	*1.82-9.83*	<0.001***
PRO(Positive)	*2.56*	*1.71-3.84*	<0.001***	*2.14*	*1.31-3.50*	0.0023**
TP	*1.02*	*1.00-1.03*	0.03*	*1.12*	*1.08-1.16*	<0.001***
ALB	1.00	0.98-1.03	0.73			
GLB	*1.02*	*1.00-1.04*	0.02*	*0.90*	*0.86-0.93*	<0.001***
T.BIL	*0.98*	*0.97-0.99*	<0.001***	1.25	0.27-5.93	0.7749
DB	*0.82*	*0.77-0.88*	<0.001***	0.65	0.13-3.18	0.5954
IB	*0.99*	*0.97-1.00*	0.02*	0.79	0.17-3.71	0.7646
ALT	*1.01*	*1.01-1.02*	<0.001***	*1.02*	*1.01-1.02*	<0.001***
AST	*1.02*	*1.01-1.02*	<0.001***	0.99	0.98-1.00	0.0873
BUN	*1.20*	*1.14-1.26*	<0.001***	0.98	0.92-1.04	0.5493
SCr	1.00	1.00-1.01	0.07			
UA	*1.00*	*1.00-1.00*	<0.001***	1.00	1.00-1.00	0.1226
TC	*1.24*	*1.17-1.31*	<0.001***	1.42	0.95-2.12	0.0859
TG	*1.51*	*1.41-1.62*	<0.001***	0.99	0.91-1.09	0.9029
HDL.C	*0.28*	*0.23-0.34*	<0.001***	*0.21*	*0.13-0.32*	<0.001***
LDL.C	*1.42*	*1.32-1.54*	<0.001***	0.87	0.54-1.39	0.5508

Statistically significant ORs and 95% CIs are shown in italics; *P <0.01, **P <0.01, ***P <0.001.

### 3.4 Analysis of independent influencing factors in the prediabetes and diabetes groups

We conducted a single-factor logistic regression analysis of the independent variables in the prediabetes and diabetes groups. Variables with P < 0.05 in the Univariate regression analysis were included in the multivariate regression analysis to analyze the factors influencing the development of diabetes in the prediabetic population. The results of the multivariate regression analysis showed that Age, BMI, SBP, U.GLU, PRO, TG, and HDL.C were independent factors influencing the development of diabetes in the prediabetic population, with Age, BMI, SBP, U.GLU, PRO, and TG being independent risk factors, and HDL.C being an independent protective factor, as shown in **Table 3**.

**Table 3:** Univariate and multivariate regression analysis for the pre-diabetes group and the diabetes group.

Variable name	Univariate regression analysis			Multivariate regression analysis		
	OR	95%CI	*P*	OR	95%CI	*P*
Age	*1.03*	*1.02-1.04*	<0.001***	*1.03*	*1.02-1.04*	<0.001***
Gender(Male)	*1.41*	*1.17-1.71*	<0.001***	1.05	0.83-1.33	0.6705
BMI	*1.06*	*1.03-1.08*	<0.001***	*1.07*	*1.04-1.1*	<0.001***
SBP	*1.02*	*1.01-1.02*	<0.001***	*1.01*	*1.00-1.01*	0.0013**
U.GLU(Positive)	*10.33*	*7.75-13.77*	<0.001***	*9.32*	*6.88-12.64*	<0.001***
PRO(Positive)	*3.21*	*2.39-4.31*	<0.001***	*2.30*	*1.65-3.22*	<0.001***
TP	0.99	0.97-1.02	0.57			
ALB	0.99	0.95-1.03	0.53			
GLB	1.00	0.97-1.02	0.84			
T.BIL	1.01	1.00-1.03	0.08			
DB	*1.12*	*1.03-1.21*	0.01*	1.01	0.92-1.12	0.7827
IB	1.01	1.00-1.03	0.15			
ALT	*1.00*	*1.00-1.00*	0.04*	1.00	1.00-1.01	0.1646
AST	1.00	1.00-1.01	0.08			
BUN	*1.15*	*1.08-1.23*	<0.001***	1.02	0.95-1.1	0.5739
SCr	1.00	0.99-1.00	0.14			
UA	1.00	1.00-1.00	0.19			
TC	*0.90*	*0.83-0.97*	0.01*	0.86	0.58-1.26	0.4305
TG	*1.12*	*1.07-1.18*	<0.001***	*1.09*	*1.02-1.16*	0.0154*
HDL.C	*0.32*	*0.23-0.45*	<0.001***	*0.43*	*0.26-0.73*	0.0016**
LDL.C	*0.89*	*0.80-0.99*	0.03*	1.34	0.85-2.11	0.2141

Statistically significant ORs and 95% CIs are shown in italics; *P <0.01, **P <0.01, ***P <0.001.

### 3.5 Construction and evaluation of a predictive model for the development of pre-diabetes in normal population

**Figure 2:**
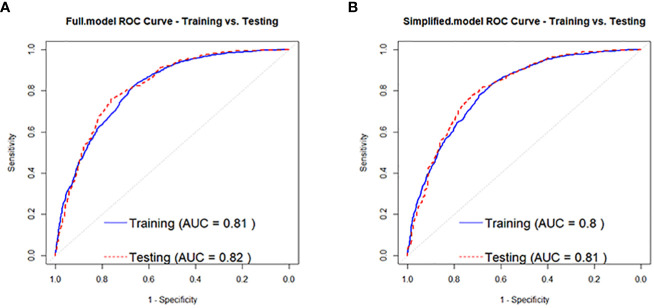
ROC curves for the normal population developing to pre-diabetes Full.model **(A)** and Simplified.model **(B)**.

According to the regression analysis results in **Table 2**, a full-variable prediction model (Full.model) was constructed for the independent risk factors that contribute to the development of prediabetes in the normal population, including Age, BMI, SBP, U.GLU, PRO, TP, GLB, ALT, and HDL.C. Based on the regression analysis results in **Tables 2**, **3**, a simplified prediction model (Simplified.model) was constructed using six variables, including Age, BMI, SBP, U.GLU, PRO, and HDL.C. The ROC curve and AUC was used to evaluate the discrimination of the predictive model, with an AUC range of 0-1, where 1 indicates complete consistency and 0.5 indicates poor consistency. The evaluation results of the models show that Full.model has a training set AUC of 0.81 (**Figure 2A**), an accuracy of 0.78, precision of 0.80, and recall of 0.89; and a training set AUC of 0.82 (**Figure 2A**), an accuracy of 0.79, precision of 0.80, and recall of 0.91. Simplified.model has a training set AUC of 0.80 (**Figure 2B**), an accuracy of 0.77, precision of 0.79, and recall of 0.89; and a training set AUC of 0.81 (**Figure 2B**), an accuracy of 0.77, precision of 0.78, and recall of 0.91.

### 3.6 Construction and evaluation of a predictive model for the progression of pre-diabetes to diabetes

**Figure 3:**
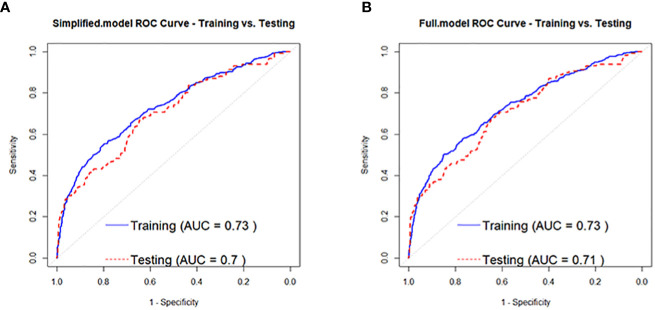
ROC curves for people with pre-diabetes developing to diabetes Full.model **(A)** and Simplified.model **(B)**.

Based on the regression analysis results in **Table 3**, a full-variable prediction model (Full.model) was constructed for independent factors predicting the development of prediabetes to diabetes in prediabetic individuals, which included Age, BMI, SBP, U.GLU, PRO, TG, and HDL.C. A simplified prediction model (Simplified.model) was also constructed using Age, BMI, SBP, U.GLU, PRO, and HDL.C as independent factors. The model evaluation results showed that Full.model had moderate discrimination for identifying high-risk individuals for developing diabetes in the prediabetic population. In the training set, the AUC of Full.model was 0.73 (**Figure 3A**), with an accuracy of 0.86, precision of 0.59, and recall of 0.20, and the AUC was 0.71 (**Figure 3A**), with an accuracy of 0.86, precision of 0.71, and recall of 0.22. Simplified.model also had moderate discrimination, with an AUC of 0.73 (**Figure 3B**), accuracy of 0.86, precision of 0.59, and recall of 0.20 in the training set, and an AUC of 0.70 (**Figure 3B**), accuracy of 0.86, precision of 0.71, and recall of 0.21.

## 4 Discussion

Diabetes has become a global public health problem, with the incidence and mortality rates increasing year by year. Prediabetes is a precursor of diabetes. Detecting prediabetes and taking effective interventions can prevent the further development of diabetes, thereby reducing the incidence and mortality rates of diabetes. In this study, we investigated the risk factors for progression from normal individuals to prediabetes and from prediabetes to diabetes, and analyzed the independent factors using multivariable logistic regression. We found that Age, BMI, SBP, U.GLU, PRO, TP, GLB, ALT, and HDL.C were independent risk factors for progression from normal individuals to prediabetes, while Age, BMI, SBP, U.GLU, PRO, TG, and HDL.C were independent risk factors for progression from prediabetes to diabetes. Among them, Age, BMI, SBP, U.GLU, and PRO were common independent risk factors for both progressions, while HDL.C was a common independent protective factor. We constructed full variable models (Full.model) and simplified models (Simplified.model) for both progressions using the above factors. The evaluation of the models indicated moderate discriminative ability and could assist in the clinical identification of individuals at high risk of developing prediabetes and diabetes.

In previous studies, many researchers have investigated the related risk factors for prediabetes and diabetes, such as Age, BMI, SBP, U.GLU, PRO, etc. Among them, Age and BMI are considered the two strongest risk factors for prediabetes (25, 26). According to estimates from the National Health and Nutrition Examination Survey (NHANES) in the United States, the overall prevalence of diabetes was 5.0% in adults under 45 years old, 17.5% in adults aged 45-64, and 33.0% in adults aged 65 and older (13). This shows that the risk of diabetes increases significantly with age. In the NHANES study, more than 80% of self-reported prediabetic patients had a BMI≥25.0, indicating that the prevalence of prediabetes is much higher in obese populations (27). A study conducted in China during a median follow-up period of 4.5 years among non-diabetic hypertensive individuals found that compared with individuals with an SBP in the range of 120-130 mmHg, those with an SBP in the range of 130-140 mmHg had a 24% increased risk of developing diabetes and a 29% reduced rate of fasting blood glucose recovery (28). Currently, it is believed that the biological mechanism between blood pressure control and the development of diabetes may be due to hypertension leading to endothelial dysfunction, which limits insulin delivery to metabolically active insulin-sensitive muscle tissue, and optimal blood pressure control can improve endothelial function and enhance microvascular perfusion, thus leading to a reduced risk of diabetes (28, 29). This study found that U.GLU positive and PRO positive in urine tests were independent risk factors for prediabetes and diabetes. These two indicators reflect the damage to the renal function of the subjects, and even if the patient’s blood glucose level returns to normal, the renal function damage caused by diabetes will continue to develop (30, 31). Studies have shown that the sensitivity of prediabetes and diabetes screening through U.GLU testing is 83.5%, and the combined use of U.GLU and FPG can significantly improve the effectiveness of diabetes screening, indicating a high correlation between U.GLU positivity and the development of diabetes (32). In any eGFR category of the general population, the incidence of diabetes and metabolic syndrome increases with increasing levels of urine protein (PRO) (33). Furthermore, studies have pointed out that observing changes in the urinary albumin-to-creatinine ratio (UACR) can predict changes in clinical outcomes and mortality risks for type 2 diabetes patients (34). Therefore, abnormal urine test results not only serve as efficient indicators for prediabetes and diabetes screening, but also serve as important indicators reflecting the level of renal function damage in diabetic patients.

However, our findings are not entirely consistent with previous studies, as we identified new indicators, including TP and ALT, as independent risk factors for the development of prediabetes in the general population. Current research has identified multiple proteins in serum that are related to the occurrence and development of prediabetes, including C-reactive protein (35), lipopolysaccharide-binding protein (36), among others. These results suggest that proteins, as the main carriers of life activities, play a complex role in the occurrence and development of prediabetes. Our study found that ALT is an independent risk factor for the development of prediabetes in the general population. Previous studies have shown that elevated ALT is associated with type 2 diabetes, indicating that ALT may be involved in insulin resistance and the development of diabetes (37, 38). Additionally, some studies have found a negative correlation between early AST/ALT levels in pregnant women and the risk of gestational diabetes mellitus (GDM), suggesting that these levels can serve as predictive factors for GDM (39). Although these findings do not directly support our results, they provide a new perspective for better understanding the occurrence and development of prediabetes.

In addition, we found that TG and LDL-C are independent risk factors for the development of diabetes in individuals with prediabetes. Our results are consistent with previous studies which have shown a positive correlation between TG and LDL-C levels and the progression of diabetes, and the predictive value of these markers for prediabetes and diabetes (40–42). Studies have also found that the TG/HDL ratio is positively correlated with β-cell dysfunction, prediabetes, and diabetes, and is an important risk assessment factor for cardiovascular disease in diabetic patients (43, 44). Campos Muniz C proposed the concept of the triglyceride glucose (TyG) index and found that it is a good predictor of DM2 (45). These findings strongly support our results and indicate that triglycerides are an important risk factor for diabetes. Abnormal blood lipids are also recognized as controllable risk factors in patients with type 2 diabetes, and their management is an important part of preventing cardiovascular disease. Studies have shown that statin therapy can significantly reduce cardiovascular events (46). Our results provide a more comprehensive and in-depth understanding of the mechanisms underlying the development of diabetes from prediabetes and provide some guidance for the diagnosis and treatment of diabetes.

This study also suggests that GLB and HDL.C are independent protective factors for the development of prediabetes in normal population, and HDL.C is an independent protective factor for the progression from prediabetes to diabetes. However, no significant evidence has been found in previous relevant studies to support the protective effect of elevated levels of GLB on the occurrence of prediabetes. A study on elderly prediabetic and elderly male populations in China found that lower levels of sex hormone-binding globulin (SHBG) were independently associated with metabolic syndrome (47). However, other studies have found that levels of alpha-fetoprotein (AFP) in gestational diabetes mellitus patients were significantly higher than in normal pregnant women, suggesting that AFP may play a role in insulin resistance and metabolic changes in gestational diabetes mellitus (48). Therefore, further research is needed on the specific association between globulin and prediabetes. HDL.C, as an independent protective factor for prediabetes and diabetes, is consistent with previous research findings (40). Studies have shown that HDL.C can not only play an anti-atherosclerotic role against endothelial cells and foam cells, but also have an anti-diabetic effect on the β-cells of the endocrine pancreas, especially by effectively inhibiting stress-induced cell death and enhancing insulin secretion stimulated by glucose (49). The increase in HDL-C levels is not only related to the reduction of cardiovascular disease risk, but also a potential strategy for preventing the occurrence and development of diabetes in the future (50). Therefore, increasing the levels of these protective factors may help prevent the occurrence of prediabetes and diabetes.

This study further constructed full variable prediction models (Full.model) and simplified prediction models (Simplified.model) for the development from normal to prediabetes and from prediabetes to diabetes, respectively, based on the independent influencing factors identified above. The model evaluations showed moderate discrimination, with the AUC of the prediabetes prediction model reaching above 0.8 and the diabetes prediction model reaching 0.7. Compared with the risk prediction models constructed by Wu et al. and Yokota et al., the discrimination of the models constructed in this study is similar, but the former two studies used blood glucose and HbA1c levels as predictors (23, 24). Blood glucose and HbA1c levels are essential for diagnosing prediabetes and diabetes and are highly correlated with the risk of developing the diseases, so the rationality of using these two indicators as predictors for constructing prediction models is questionable. The Simplified.model constructed in this study by simplifying the variables has moderate discrimination for identifying prediabetes and diabetes, and the included indicators are commonly used in clinical physical examinations. Therefore, it can be considered that the model has certain applicability and is expected to contribute to the prevention and management of prediabetes and diabetes in the future.

In previous studies, several diabetes prediction models have been developed using statistical models such as logistic regression, Cox proportional hazards model, or Weibull distribution analysis. The predictive accuracy of these traditional statistical methods, as measured by the C-index, ranged from approximately 0.74 to 0.94. In recent years, the development of artificial intelligence (AI) technology has presented new opportunities and challenges for diabetes prevention, diagnosis, and treatment. AI’s main applications in diabetes include automated retinal screening, clinical diagnostic support, patient self-management tools, and risk stratification (51, 52). Currently, the aggregated AUC (Area Under the Curve) of artificial intelligence in diabetes prediction and risk stratification is approximately 0.86-0.88, indicating a high level of discrimination ability (53, 54). However, it is premature to conclude that machine learning surpasses traditional statistical analysis in predicting incident diabetes in specific populations. Furthermore, AI-generated models may suffer from overfitting, leading to highly accurate predictions for the training population but significantly reduced accuracy when applied to the validation population. Although there are still challenges in using machine learning models to predict incident diabetes in clinical practice, we firmly believe that in the future, more efficient machine learning models and the availability of larger omics databases will undoubtedly contribute to further improving prediction accuracy.

This study used logistic regression analysis to identify the independent risk factors for prediabetes and diabetes, most of which were independent risk factors. The results of this study help to strengthen people’s awareness and control of the risk factors for diabetes, and take proactive measures to control the relevant factors for prediabetes and diabetes, which is of great significance for the prevention and control of diabetes. Furthermore, the predictive models constructed based on the results of this study can help identify high-risk individuals for prediabetes and diabetes.

This study still has several limitations. Firstly, all samples were obtained from a single hospital, and the sample size was relatively small. Additionally, the study subjects were all from the same region, which may lead to biases and lack of representativeness, making it challenging to generalize the research findings. Secondly, due to inadequate data collection, some crucial variables (such as medications taken by participants, ethnicity, lifestyle patterns, sleep habits, and smoking status) were not investigated. Including these variables could significantly enhance the impact of the research results. Furthermore, this study was cross-sectional and employed logistic regression analysis, which can identify independent influencing factors but cannot establish causal relationships. Lastly, the predictive model constructed in this study was only internally validated and lacked external validation and real-world application research.

Hence, future research could consider enlarging the sample size and adopting a multi-center research design to improve the reliability of the conclusions. Additionally, more scientifically rigorous study designs, such as cohort studies and randomized controlled trials, could be employed to further assess causality and explore comprehensive diabetes risk factors. Ultimately, more advanced algorithms should be considered to develop predictive models with better discrimination and evaluation, which can be applied to the identification of high-risk populations in the real world.

## 5 Conclusion

This study found that Age, BMI, SBP, U.GLU, PRO, TP, and ALT were independent risk factors, while GLB and HDL.C were independent protective factors for developing prediabetes from the normal population. For those who progressed from prediabetes to diabetes, Age, BMI, SBP, U.GLU, PRO, and TG were independent risk factors, while HDL.C was an independent protective factor. By including these factors as predictors, a prediction model was developed that had moderate discriminative ability for identifying individuals at high risk for prediabetes and diabetes. In this study, blood glucose and HbA1c were used as the diagnostic gold standards, while other risk indicators were considered as predictive factors. This approach allows for the identification and early warning of individuals at risk of prediabetes and diabetes among the population undergoing health examinations.

## Data availability statement

The original contributions presented in the study are included in the article/**Supplementary Material**. Further inquiries can be directed to the corresponding authors.

## Ethics statement

The studies involving humans were approved by Peking University Shenzhen Hospital Ethical Review Committee. The studies were conducted in accordance with the local legislation and institutional requirements. Written informed consent for participation in this study was provided by the participants’ legal guardians/next of kin. Written informed consent was obtained from the individual(s) for the publication of any potentially identifiable images or data included in this article.

## Author contributions

DG, XC and LY acquired, analyzed, discussed the data and drafted the manuscript. DG, YZ, JL and CY analyzed and discussed the data. YC, WY and JW designed the research and revised the manuscript. All authors contributed to the article and approved the submitted version.
